# Newborn Hospitalizations Before and During COVID-19 Pandemic in Poland: A Comparative Study Based on a National Hospital Registry

**DOI:** 10.3389/ijph.2024.1606272

**Published:** 2024-02-14

**Authors:** Krzysztof Kanecki, Katarzyna Lewtak, Piotr Tyszko, Irena Kosińska, Patryk Tarka, Paweł Goryński, Aneta Nitsch-Osuch

**Affiliations:** ^1^ Department of Social Medicine and Public Health, Medical University of Warsaw, Faculty of Medicine, Warsaw, Poland; ^2^ Institute of Rural Health in Lublin, Lublin, Poland; ^3^ Department of Population Health Monitoring and Analysis, National Institute of Public Health NIH—National Research Institute, Warsaw, Poland

**Keywords:** epidemiology, hospitalization, newborns, COVID-19, in-hospital fatality, nationwide database, Poland

## Abstract

**Objectives:** There are limited data on the impact of the COVID-19 outbreak in Poland on newborn health. The aim of the study is to show recent information on hospitalizations of newborns in Poland in the pre-pandemic and COVID-19 pandemic era.

**Methods:** A retrospective, population-based study was conducted using data from hospital discharge records of patients hospitalized in 2017–2021.

**Results:** The data on which the study was based consisted of a substantial number of 104,450 hospitalization records. Annual hospitalization rate was estimated to be 50.3–51.9 per 1,000 in 2017–2019, 56 per 1,000 in 2020 and it rose to 77.7 per 1,000 in 2021. In comparison to the pre-pandemic period, in the COVID-19 era, we observed significantly more hospitalization cases of newborns affected by maternal renal and urinary tract diseases (*p* < 0.001), syndrome of infant of mother with gestational diabetes (*p* < 0.001), maternal complications of pregnancy (*p* < 0.001). In the COVID-19 era, the prevalence of COVID-19 among newborns was 4.5 cases per 1,000 newborn hospitalizations.

**Conclusion:** The COVID-19 pandemic outbreak could significantly contribute to qualitative and quantitative changes in hospitalizations among newborns.

## Introduction

The COVID-19 pandemic affected populations and healthcare systems worldwide and may impact newborn health. A study on approx. 400,000 women with symptomatic COVID-19 who were 15–44 years of age showed that pregnant women were more likely than nonpregnant women to be at risk of intensive care unit admission, invasive ventilation, extracorporeal membrane oxygenation and death [1]. It was reported that maternal outcomes deteriorated in the COVID-19 era globally which was confirmed by the observed rise in maternal depression, ruptured ectopic pregnancies, stillbirth and maternal deaths [2]. All-cause mortality in pregnant patients was 11.3% whereas the case fatality rate in the group of non-pregnant hospital patients was estimated at 6.4% [3]. Studies comparing pregnant women with severe and non-severe COVID-19 showed that women with severe COVID-19 were 3.7 years older and the risk of severe COVID-19 was 1.5 times higher among women >35 years. Obese women, smokers, diabetics and those who had pre-eclampsia were at an increased risk of developing severe COVID-19 [4]. However, a cohort study on pregnant women with COVID-19 that was conducted by the World Health Organization showed that only 8% and 1% of the women were severely and critically ill, respectively [5]. Interestingly, pregnant women with COVID-19-related pneumonia showed a milder course of disease and good recovery [6], and what is more, mortality in this group of patients was lower than in the general group of COVID-19 patients [7]. It seems that SARS-CoV-2 positivity in a child can affect the severity of maternal infections [8].

No evidence was found either for vertical transmission of the virus in the late stages of pregnancy [9]. However, in one study, the vertical transmission rate was calculated using SARS-CoV-2 nucleic acid tests and it accounted for 3.91% [7]. Nevertheless, other studies that examined samples of amniotic fluid, umbilical cord, placenta and breast milk all tested negative, except for one sample of amniotic fluid [10]. An analysis of data on 45 children born to mothers with SARS-CoV-2 revealed 3 positive newborns (6.6%); however, a throat swab was used for this test and a negative result was obtained over time, which may be indicative of transient colonization [11].

When compared with children born to mothers without COVID-19, those whose mothers were diagnosed with COVID-19 showed significantly increased severe neonatal morbidity index, and severe perinatal morbidity and mortality index [12]. Pregnant women with diagnosed COVID-19 are more likely to deliver preterm and their newborns are more likely to be admitted to the neonatal unit than those born to mothers without COVID-19 [13]. Pregnant women with comorbidities were at an increased risk for severe outcomes related to COVID-19, adverse birth outcomes and maternal morbidities [14]. Regarding the adverse outcomes in newborns, these may be associated with maternal diseases rather than COVID-19 infections, especially in the subgroup of children born to critically ill mothers [15]. A significant proportion of placentas of pregnant women with COVID-19 showed histopathologic findings, which indicated placental hypoperfusion and inflammation [16]. A multicenter cohort study revealed the following rates in a group of pregnant women diagnosed with COVID-19 using an RT-PCR test: cesarean section 71.2%, prematurity 26.4%, and low-birthweight infant rates 12.8% [17]. The route of delivery may be associated with the risk of infection in newborns. A review of newborns whose mothers were infected with COVID-19 showed that 2.7% of those born vaginally were tested positive as compared with 5.3% of those born by C-section [18]. Pooled proportions and a higher rate of preterm birth, preeclampsia, Cesarean section, and perinatal death were associated with COVID-19 infection [19]. In another study, the risk of preterm birth in women with severe COVID-19 was assessed as 2.4 fold higher than in the group of pregnant women without COVID-19 [4].

There are limited data on the impact of the COVID-19 outbreak in Poland on newborn health. The aim of the study is to show recent information on factors related to hospitalizations of newborns in Poland in the pre-pandemic and COVID-19 pandemic era.

## Methods

### Data Collection

A retrospective, population-based study was conducted using data from 104,450 hospital discharge records of patients hospitalized in 2017–2021 that were registered in the Nationwide General Hospital Morbidity Study (NGHMS) kept by the National Institute of Public Health in Poland and the data covered hospitalization records of newborns requiring in-hospital care immediately after birth. NGHMS is conducted within the framework of the Programme of Statistical Surveys of Official Statistics. All Polish hospitals, except for psychiatric facilities, are legally bound to provide hospital discharge data to the Institute. These are anonymous data on the patient’s sex, date of birth and place of residence, hospital admission and discharge and the discharge diagnostic codes according to the International Classification of Diseases and Related Health Problems 10th Revision (ICD-10).

The inclusion criterion was immediate hospitalization on the day of birth and hospitalization with the primary or secondary ICD-10 codes of Certain conditions originating in the perinatal period and hospitalized immediately after birth. In the study, a comparison analysis was made between hospitalizations in the pre-pandemic period, i.e., before 4 March 2020, and the COVID-19 era. The first case of COVID-19 in Poland was reported on 4 March 2020. Diseases that occurred in the study group more frequently than 5% were included in the analysis. More than 99% of newborns in the study population presented at least one of the reported diseases.

### Statistical Analysis

To perform the statistical analyses, Statistica (TIBCO Software Inc., Palo Alto, California, United States) [20] and WINPEPI [21] were used. The following statistical measures were computed: means, medians, and ranges for continuous variables, counts and percentages for categorical variables. For continuous variables with normal or non-normal distribution, respectively, means and 95% confidence intervals or medians and interquartile ranges were calculated. For nominal variables, counts and percentages were analyzed. Rates of hospitalizations were calculated as the estimated number of cases per 1,000 newborns using data (national census) from Statistics Poland [22]. Analyses were performed using the t-Student test with respect to an assumption of normal distribution in sufficiently large samples in public health research [23]. When normality assumptions were not met, non-parametric tests were applied. A two-sided *p*-value lesser than 0.05 was considered to be statistically significant.

### Ethical Approval

As the data were anonymous, and they did not include personal data or other data which could be used to identify patients therefore waiver for ethical approval and informed consent is granted by the National Institute of Public Health NIH—National Research Institute Bioethics Committee.

## Results

Number of hospitalizations of newborns and all hospitalizations in Poland per year in 2017–2021 are presented in Table 1. Annual hospitalization rate was estimated to be 50.3–51.9 per 1,000 in 2017–2019, 56 per 1,000 in 2020 and it rose to 77.7 per 1,000 in 2021. We observed an increased percentage of newborn hospitalizations in 2021 when compared to 2020 and the 2017–2019 period. The study group consisted of 53,782 male newborns (51.5% of all patients) and 50,660 female newborns (48.5% of all patients), in eight cases sex was not specified. The mean and median length of hospitalization stay during the entire study period (2017–2021) were 4.33 (95% CI: 4.3–4.35) days and 3 (IQR 3–5) days, respectively. The mean hospitalization rate in 2017–2019 accounted for 50.5 per 1,000 newborns (95% CI: 47.2–53.8), and it rose to 56 per 1,000 in 2020. The highest rate was 77.7 per 1,000 in 2021, which was significantly higher when compared to the 2017–2019 period (*p* < 0.001).

**TABLE 1:** Hospitalization of newborns and all hospitalizations in Poland, 2017–2021. Nationwide General Hospital Morbidity Study, Poland 2017–2021 (Poland, 2023).

	Years				
	2017	2018	2019	2020	2021
Number of hospitalization in the study—total	20,207	19,136	19,471	19,886	25,750
Percentage in relation to all newborns in Poland	5.0%	4.9%	5.2%	5.6%	7.8%
All newborns in Poland—total	401,982	388,178	374,954	355,309	331,511
Percentage in relation to all hospitalizations in Poland	0.27%	0.25%	0.26%	0.36%	0.40%
All hospitalizations in Poland—total	7,574,153	7,612,875	7,541,382	5,546,387	6,465,539

In our study, we found 62,709 hospitalization records in the pre-pandemic period (60% of all cases) and 41,741 records in the COVID-19 era (40% of all cases). Statistically significant differences in the occurrence of selected diseases among newborns in the pre-pandemic era and the COVID-19 era were observed, as presented in Table 2. In most cases, we reported a significant increase in the percentage of the disease, however, a significantly lower percentage of jaundice was observed in the study.

**TABLE 2:** Certain conditions originating in the perinatal period in pre- and pandemic period. Nationwide General Hospital Morbidity Study, Poland 2017–2021 (Poland, 2023).

Certain conditions originating in the perinatal period (ICD10-codes)	Pre-pandemic—total number (cases per 1,000 newborns)	Pandemic—total number and (cases per 1,000 newborns)	Pandemic/pre-pandemic—ratio (%)	*P*
Newborn affected by maternal conditions that may be unrelated to present pregnancy (P00)	62,686 (999.63)	41,719 (999.47)	99.98	0.221
Newborn affected by maternal hypertensive disorders (P00.0)	12,025 (191.76)	8,029 (192.35)	100.31	0.811
Newborn affected by maternal renal and urinary tract diseases (P00.1)	8,569 (136.65)	6,661 (159.58)	116.78	*p* < 0.001
Newborn affected by maternal infectious and parasitic diseases (P00.2)	36,582 (583.36)	24,417 (584.96)	100.27	0.607
Newborn affected by other maternal conditions (P00.8)	5,536 (88.28)	3,654 (87.54)	99.16	0.679
Newborn affected by maternal complications of pregnancy (P01)	2,959 (47.19)	2,196 (52.61)	111.49	*p* < 0.001
Newborn affected by complications of placenta, cord and membranes (P02)	2,674 (42.64)	2,727 (65.33)	153.21	*p* < 0.001
Newborn affected by other compression of umbilical cord (P02.5)	1,906 (30.39)	2,272 (54.43)	179.08	*p* < 0.001
Disorders of newborn related to short gestation and low birth weight, not elsewhere classified (P07)	4,758 (75.87)	3,150 (75.47)	99.46	0.807
Preterm (premature] newborn (other] (P07.3)	3,955 (63.07)	2,585 (61.93)	98.19	0.456
Extreme immaturity of newborn (P07.2) or preterm (premature] newborn (other] (P07.3)	4,033 (64.31)	2,631 (63.03)	98.01	0.407
Neonatal jaundice from other and unspecified causes (P59)	7,745 (123.51)	4,679 (112.1)	90.76	*p* < 0.001
Neonatal jaundice, unspecified (P59.9)	6,470 (103.18)	3,838 (91.95)	89.12	*p* < 0.001
Transitory disorders of carbohydrate metabolism specific to newborn (P70)	5,432 (86.62)	4,637 (111.09)	128.25	*p* < 0.001
Syndrome of infant of mother with gestational diabetes (P70.0)	4,145 (66.10)	3,791 (90.82)	137.4	*p* < 0.001

In our study, in the COVID-19 era, 187 newborns with ICD10 codes reporting the presence of COVID-19 were observed (94 males, 93 females) and the prevalence of COVID-19 accounted for 4.5 cases per 1,000 hospitalizations. In the group of 187 newborns with COVID-19, 17 patients, were premature babies, 153 were not premature babies, and with regard to 17 patients there was no information in this respect. In our study, we did not observe significant differences in the percentage of all premature newborns between the pre-pandemic and the COVID-19 era. However, in the COVID-19 era, we observed a significantly higher percentage of premature in newborns with COVID-19 in comparison to the newborns without COVID-19 (9.1% vs. 5.7%, *p* < 0.05). No COVID-19 newborns died in hospital.

**FIGURE 1:**
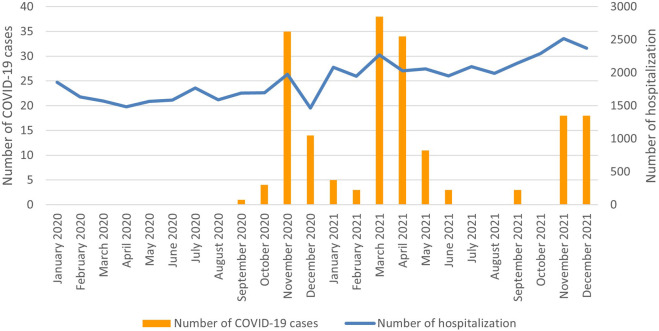
Number of hospitalization and number of hospitalized newborns with COVID-19 per month, 2020–2021 (Poland, 2023).

A seasonal pattern of COVID-19 hospitalizations of newborns was observed, as shown in Figure 1.

In the study period, significantly more newborns were from urban than rural regions (62% vs. 38%; *p* < 0.001). However, in the COVID-19 era, newborns from rural regions were more often hospitalized in comparison to the pre-pandemic period (41.06% vs. 40,26%; *p* < 0.02).

During the observation period, 104 deaths were reported (0.1% of all patients), 48 of which occurred in males, and 56 in females. In urban regions, we observed 64 cases of death (61.5%), in rural regions 37 cases (35.6%), and with regard to 3 cases (2.9%), the place of living was not specified as rural or urban. In the pre-pandemic era, we observed 63 fatal hospitalizations, as compared to 41 cases in the COVID-19 era, and there were no significant differences in the rate of fatal hospitalizations in the post-pandemic period in relation to the pre-pandemic period. The most often reported underlying causes of death included anencephaly (Q00.0 ICD10 codes) which was observed in 29 cases (27.9% of all deaths) among them 11 cases were in the pre-pandemic era, 18 cases in the COVID-19 era. Extreme immaturity of newborn (P07.2 ICD10 codes) was found in 13 cases (12.5% of all cases) among them 9 cases were in the pre-pandemic era, and 4 cases in the COVID-19 era. Other causes were below 6%, separately.

## Discussion

Although the overall number of births in Poland is gradually decreasing, as presented in Table 1, a significant increase in neonatal hospitalizations was observed in 2021 as compared to 2017–2019 and 2020 when the COVID-19 pandemic outbreak began in Poland. The total number of hospitalizations in Poland in 2021 was higher than in 2020, but lower than in 2017–2019, as presented in Table 1. Organizational changes in healthcare after the COVID-19 outbreak in Poland may be a factor contributing to the decrease in all hospitalizations in 2020–2021 in Poland; however, we observed an increased percentage of hospitalizations of newborns in 2020–2021. The increase in hospitalization in 2021 may be associated with changes in the public’s awareness of the risks associated with COVID-19. Additionally, in the era of the COVID-19 pandemic, a significant proportion of pregnant women may have been infected with asymptomatic or symptomatic COVID-19 or a history of SARS-Cov-2 infection in the mother could have contributed to the necessity of a newborn’s hospitalization after birth.

In the subgroup of newborns with COVID-19, we observed a periodic variability in the number of hospitalizations, as presented in Figure 1. Variations in the incidence of COVID-19 infections could also be observed in the general population of Poland [24]. This observation may suggest a relationship between the incidence of COVID-19 infections among newborns and COVID-19 infections in the general population.

In the COVID-19 era, newborns from rural regions were more often hospitalized in comparison to the pre-pandemic period. Limitations in access to healthcare for pregnant women may be greater for people living in rural areas. A retrospective cohort study from the United States that was based on a large set of data of 1,033,229 patients showed that hospitalization, inpatient mortality, and other adverse outcomes are greater in the group of rural patients with COVID-19 than in their urban counterparts [25]. A possible consequence of restrictions for pregnant women may be a relatively higher rate of hospitalization of newborns from rural areas, which was observed in this study.

We found significant differences in the incidence of certain diseases and we did not see any significant differences in the case of other selected diseases, as reported in Table 2. It can be assumed that both the COVID-19 pandemic and subsequent healthcare organizational changes implemented after the outbreak of the pandemic may have contributed to changes in morbidity among pregnant women. Interestingly, some of the diseases reported in newborns may relate to the health of women during pregnancy. It was observed that pregnant women with underlying diseases were more likely to suffer from COVID-19 than other patients, and those infected showed an increased rate of preterm birth [26]. However, when we compared the pre-pandemic period and the COVID-19 era, the rate of preterm births did not differ significantly. A recent study showed that some factors, diabetes mellitus in particular, were predictive of poor pregnancy outcomes in patients with COVID-19 [27]. In a large-scale study on 128,176 non-pregnant and 10,000 pregnant patients with confirmed COVID-19, diabetes turned out to be the most common comorbidity in pregnant patients (18%) [3]. However, current evidence shows that COVID-19 in pregnancy rarely affects fetal and neonatal mortality, although it can be associated with adverse neonatal morbidities [28]. Interestingly, a significantly lower incidence of jaundice was observed in the study; however, further studies are necessary for better analysis of these observations.

In our study, we did not observe significant changes in the incidence of preterm birth among newborns after the outbreak of the pandemic. The COVID-19 pandemic was suggested to be associated with preterm birth [29]. Increased awareness of pregnant women, changes in obstetrics and gynecology care or other organizational activities in public health could probably play an important role in hospitalizations of premature infants. One of the important factors that may contribute to changing the outcome of newborns after birth may be the use of COVID-19 vaccinations. COVID-19 vaccination in the pregnancy period was related to a decreased risk of neonatal intensive care unit admission, intrauterine fetal death, and maternal SARS-CoV-2 infection [30].

In our study, we observed an increased percentage of premature newborns among COVID-19 newborns after the COVID-19 outbreak in Poland, in comparison to the newborns without COVID-19. We had no data on the prevalence of COVID-19 among pregnant women in our study. However, a systematic review of pregnant women with COVID-19 and their neonates revealed 65 (23.6%) preterm neonates [10]. It was also reported that COVID-19 infection in pregnancy could lead to an increased risk in pregnancy-related complications [31]. Another study showed that SARS-CoV-2 infection during pregnancy could be associated with preterm birth [32]. It was reported that newborns of mothers infected with SARS-CoV-2 were at an increased risk of preterm birth and admission to the Neonatal Intensive Care Unit [33]. Low birth weight and preterm birth were more likely to develop in pregnant women with COVID-19 than in those without COVID-19 [3].

In our study, a low percentage of newborns diagnosed with COVID-19 infections was observed. The majority of neonates with SARS-CoV-2 infection were asymptomatic or showed mild symptoms [34]. A large meta-analysis revealed that most neonates born to infected mothers showed no clinical abnormalities (80.4%) and 10.0% of those tested were positive [35]. Another study revealed the pooled percentage of infected neonates at 6.0% [36]. Yet another study showed the rate of positive SARS-CoV-2 tests in neonates born to mothers with COVID-19 at 8% [3].

During the observation period, only 0.1% of all hospitalization cases were fatal, and none of the newborns with COVID-19, who were subject to our study, died. Mortality in neonates with positive RT-PCR test was reported to be 1.7% in other studies, the prognosis was good, and mortality was mainly associated with other comorbidities [37]. In another study, neonatal mortality ranged from 2% in case reports to 3.3% in case series [38]. SARS-CoV-2 infection at any stage of pregnancy was found to raise the risk of neonatal morbidity, but it did not affect stillbirth or intrauterine growth restriction [39]. Another study showed that pregnant women with positive SARS-CoV-2 test results had higher odds of stillbirths and perinatal mortality [40].

### Strengths and Limitations

Our study has certain limitations. The analyzed hospital discharge database did not include other variables related to pregnant women which might be related to the hospitalization of newborns. The study did not analyze the reliability or completeness of the reported data; however, the obligation to report hospitalization data to the database used in this study results from the law, and the rules of public statistics. Considering the study results, it seems reasonable to search for an explanation for the increase in neonatal hospitalizations with regard to the factors of maternal health and healthcare.

### Conclusion

The COVID-19 pandemic outbreak could have significantly contributed to qualitative and quantitative changes in hospital care among newborns and increased the need for healthcare among newborns. The results of this study may be helpful in comparative analyses in the European and global context and may be helpful in building targeted and effective activities limiting the consequences of the COVID-19 outbreak. Further research on newborns is needed, including data on maternal morbidity during pregnancy.
